# OP58 Cost Utility Of Ataluren Plus Standard Care In Patients With Nonsense Mutations Duchenne Muscular Dystrophy In Brazil

**DOI:** 10.1017/S0266462325101165

**Published:** 2025-12-29

**Authors:** Eliane Würdig Roesch, Verônica Colpani, Grace Anne Dória, Klébya Hellen Dantas de Oliveira, Catia Oliveira, Henry Maia Peixoto, Ana Flávia de Morais Oliveira

## Abstract

**Introduction:**

Duchenne muscular dystrophy (DMD) is a rare, X-linked recessive disease in which dystrophin deficiency leads to progressive muscular degeneration. Ataluren is a mutation-specific therapy that delays disease progression. Our study aimed to assess the cost-utility of ataluren plus standard care (SC), compared with SC alone, in patients with nonsense mutations DMD from a Brazilian Unified Health System perspective.

**Methods:**

A semi-Markov model with five health states based on ambulatory status was developed to assess the incremental cost-effectiveness ratio (ICER) of ataluren plus SC versus SC alone, which was expressed as BRL per quality-adjusted life year (QALYs). A 50-year time horizon was used in the base case analysis, considering annual cycles. Both costs and QALYs were discounted five percent. Transition probabilities and utility data were taken from published literature. Costs were based on data from a micro-costing study and the Brazilian Chamber of Drug Market Regulation database. Parameter uncertainties were tested using deterministic and probabilistic sensitivity analysis.

**Results:**

Ataluren plus SC led to incremental 0.503 QALYs and incremental costs of BRL32,453,742.94 (USD13,298,877.45), resulting in an ICER of BRL64,463,723.25 (USD26,415,910.08) per QALY. The deterministic sensitivity analysis showed that early ambulatory state utility was the most significant for the ICER, followed by costs of ataluren plus SC related to early ambulatory state. All estimates in the probabilistic sensitivity analysis were above the willingness-to-pay threshold of BRL120,000.00 (USD49,180.33) per QALY, which takes into consideration the rarity of the disease.

**Conclusions:**

The ICER value was 537 times greater than the cost-effectiveness threshold of BRL120,000.00 (USD49,180.33). Therefore, ataluren plus SC is not a cost-effective therapy for patients with nonsense mutations DMD. However, it is essential to discuss cost-effectiveness threshold, value-based prices, and commercial agreement for pricing of orphan drugs in order to provide more effective therapies and prevent judicialization.

